# Transmural variations of vasodilator-induced changes of myocardial oxygenation vary with age and the presence of diabetes mellitus type II: a study using oxygen-sensitive cardiovascular MR

**DOI:** 10.1186/1532-429X-15-S1-P132

**Published:** 2013-01-30

**Authors:** Judy Luu, Anna Schmidt, Jacqueline Flewitt, Andrew G Howarth, Matthias G Friedrich

**Affiliations:** 1 Cardiac Sciences, University of Calgary, Calgary, AB, Canada; 2 Montreal Heart Institute, Montreal, QC, Canada

## Background

Diabetes is a chronic disease process, which precedes the development of microvascular dysfunction, detectable as circumferential, subendocardial perfusion deficits on imaging. The full effect of this chronic disease process on myocardial oxygenation is, however, unknown. Studies using oxygen-sensitive Cardiovascular Magnetic Resonance (OS-CMR) have shown that pharmacologically induced vasodilation leads to an increase of myocardial oxygenation. We hypothesized that in patients with type 2 diabetes, there is an impaired increase of myocardial oxygenation.

## Methods

We measured the relative change of myocardial oxygenation during adenosine infusion in eight patients with type 2 diabetes (53±7 yrs, 6 male) and compared results to 11 healthy volunteers (29±4 yrs, 6 male), and 17 controls matched for age, gender and BMI (51±10 yrs, 13 male), all with no history of ischemic heart disease. Using a clinical 1.5T MRI system, OS-CMR was performed at baseline and during adenosine infusion (140 μg/kg/min) in three short axis slices in the study group and a single, mid-ventricular slice for the healthy volunteers. Myocardial signal intensity (SI) was measured in the whole myocardium, as well as separately in subepicardial and subendocardial layers.

## Results

**Figure 1:**
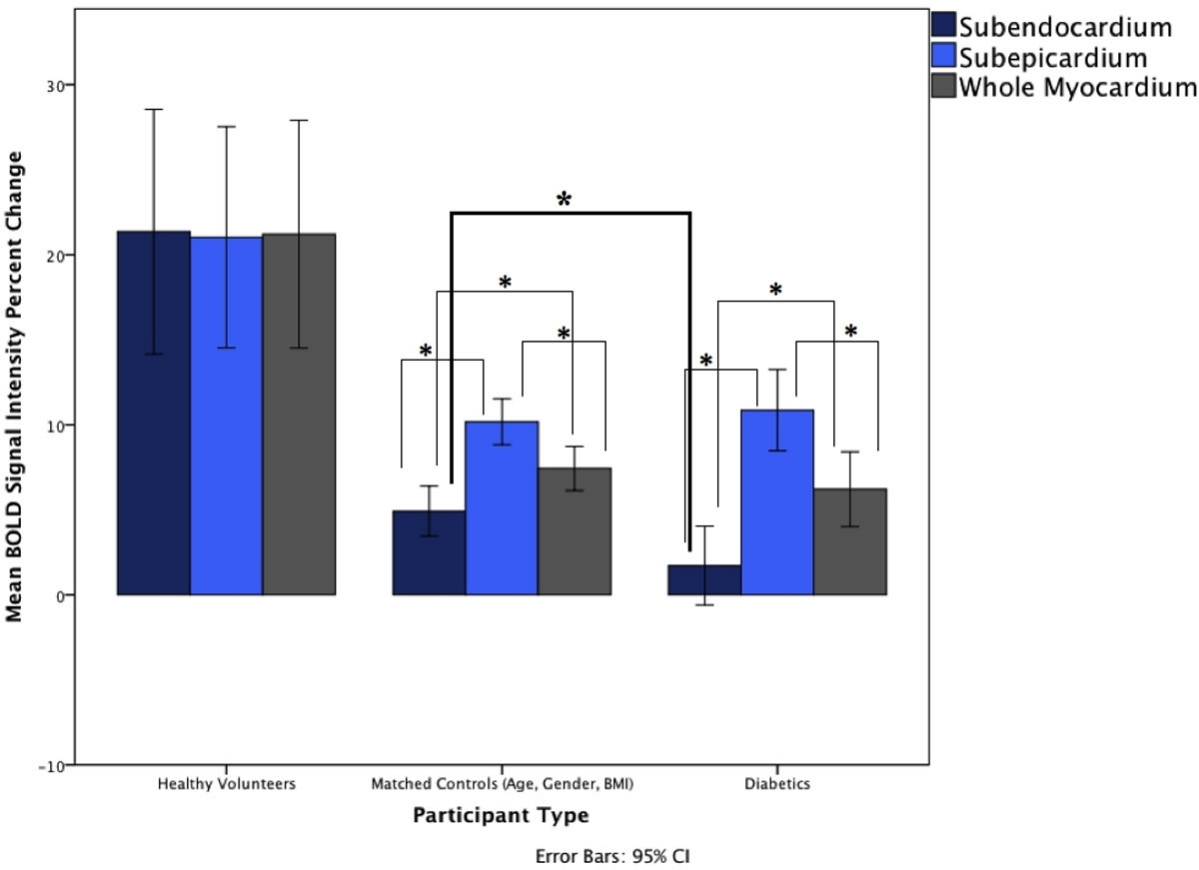
Mean BOLD signal intensity percent changes in the whole myocardium, subendocardium, and subepicardium in study participants. Significance (*) indicated at p<0.05.

A total of 66 segments from healthy volunteers, 211 segments from the diabetic patients and 100 segments from the matched controls were analyzed. The percent change between baseline and hyperemia is presented in Table 1. Diabetic patients and matched controls had an overall reduced response to hyperemia across the whole myocardium when compared to young healthy subjects. There was no detectable transmural gradient of the response in healthy subjects, whereas diabetic patients had a significantly reduced response in the subendocardium when compared to the subendocardial segments of matched controls (Figure 1).

**Table 1:** Mean BOLD signal intensity percent changes for the whole myocardium, subendocardium, and subepicardium in study participants.

	Whole myocardial SI Δ	Subendocardial SI Δ	Subepicardial SI Δ
Healthy volunteers (n=11 patients; 66 segments)	21.19% (95% CI 14.51 to 27.88)	21.34% (95% CI 14.15 to 28.53)	21.02% (95% CI 14.51 to 27.52)

Matched controls (age, gender, BMI) (n=17 patients; 211 segments)	7.42% (95% CI 6.12 to 8.71)	4.92% (95% CI 3.44 to 6.39)	10.17% (95% CI 8.82 to 11.51)

Diabetics (n=8; 100 segments)	6.21% (95% CI 4.01 to 8.40)	1.71% (95% CI -0.61 to 4.03)	10.85% (95% CI 8.46 to 13.22)

p Value	p=0.000	p=0.000	p=0.005

## Conclusions

Compared with a healthy and young population, middle-aged subjects demonstrate a transmural gradient of vasodilator-induced changes of myocardial oxygenation. Compared with matched controls, however, patients with diabetes demonstrate a more severely impaired subendocardial response. Oxygen-sensitive CMR may serve as a valuable tool to non-invasively detect microvascular dysfunction in diabetes.

## Funding

Canadian Diabetes Association; Alberta Innovates Health Solutions

